# RNA Vaccines: Yeast as a Novel Antigen Vehicle

**DOI:** 10.3390/vaccines11081334

**Published:** 2023-08-07

**Authors:** Anna Jéssica Duarte Silva, Mylenna Máyra Gois de Sousa, Larissa Silva de Macêdo, Pedro Luiz de França Neto, Ingrid Andrêssa de Moura, Benigno Cristofer Flores Espinoza, Maria Da Conceição Viana Invenção, Samara Sousa de Pinho, Marco Antonio Turiah Machado da Gama, Antonio Carlos de Freitas

**Affiliations:** Laboratory of Molecular Studies and Experimental Therapy—LEMTE, Department of Genetics, Federal University of Pernambuco, Recife 50670-901, Brazil; anna.jessica@ufpe.br (A.J.D.S.);

**Keywords:** therapeutic vaccines, mRNA vaccines, antigenic delivery, whole yeast vaccine, delivery vehicle

## Abstract

In the last decades, technological advances for RNA manipulation enabled and expanded its application in vaccine development. This approach comprises synthetic single-stranded mRNA molecules that direct the translation of the antigen responsible for activating the desired immune response. The success of RNA vaccines depends on the delivery vehicle. Among the systems, yeasts emerge as a new approach, already employed to deliver protein antigens, with efficacy demonstrated through preclinical and clinical trials. β-glucans and mannans in their walls are responsible for the adjuvant property of this system. Yeast β-glucan capsules, microparticles, and nanoparticles can modulate immune responses and have a high capacity to carry nucleic acids, with bioavailability upon oral immunization and targeting to receptors present in antigen-presenting cells (APCs). In addition, yeasts are suitable vehicles for the protection and specific delivery of therapeutic vaccines based on RNAi. Compared to protein antigens, the use of yeast for DNA or RNA vaccine delivery is less established and has fewer studies, most of them in the preclinical phase. Here, we present an overview of the attributes of yeast or its derivatives for the delivery of RNA-based vaccines, discussing the current challenges and prospects of this promising strategy.

## 1. Introduction

Vaccination is the most effective prophylaxis measure for infectious diseases [1]. According to the WHO, the eradication of diseases, such as poliomyelitis, and the reduction in mortality of others such as tetanus, whooping cough, and influenza, was only achieved thanks to vaccines. However, there are still some obstacles, such as keeping up with the changes caused by genetic variants and generating more effective responses, in addition to the slow production of some types of immunogen [2].

Within this scenario, the mRNA-based vaccine production strategy proved to be a safe, fast, and effective alternative. Primarily developed decades ago, RNA vaccines took a long time to be widely employed due to inherent problems such as low stability, excessive immunostimulation, and low efficacy in some trials [3,4]. However, with advances in sequence modifications, such as codon optimization, chemical modifications, and the use of carrier molecules, the possibility of their use in clinical applications has grown [5].

In parallel, the interest in more efficient delivery systems has also received attention. Several methods have already been adopted, such as lipid nanoparticles used in mRNA vaccines developed for SARS-CoV-2 [6,7]. However, the application of microorganisms as delivery systems has been gaining prominence due to their ability to generate efficient immune responses, specific capture, and physical protection for the vaccine antigen [8]. In this context, yeasts such as *Saccharomyces cerevisiae*, *Schizosaccharomyces pombe*, and *Pichia pastoris* have been proposed as promising delivery systems for acid nucleic-based vaccines [9,10]. Among the aspects that make yeasts good options for gene delivery are their efficiency in activating human and murine immune cells, their safety for administration, and their recognized use as a delivery system for protein antigens [9,11]. Yeasts do not affect the viability of APCs after phagocytosis, unlike other organisms, such as bacteria, which can be cytotoxic, they are particulate natural adjuvants, and protect the vaccine antigen from biodegradation [12,13,14,15,16]. In vivo studies have employed different routes of administration: oral, intramuscular, intraperitoneal, and subcutaneous [10]. The carrying of nucleic acids by yeast allows the exploration of routes for immunization that would be unfeasible in the absence of a delivery system or influenced by the composition of the carrier. Overall, yeast-based vaccines are being evaluated in different animal models such as mice, fish, pigs, and chickens [9]. Although the whole yeast vaccine (WYV) is not yet commercially available, some therapeutic vaccines for cancer and chronic diseases are already in the clinical evaluation phase [17,18,19]. Unlike yeast vaccines carrying protein antigens, the WYV for DNA or RNA vaccine delivery has fewer in vivo studies, all of them performed in an animal model. In this review, we describe how yeasts can be used as an antigen vehicle to enhance the effectiveness of RNA vaccines, and the main applications in immunotherapy approaches.

## 2. RNA Vaccines and Delivery Systems

**Figure 1:**
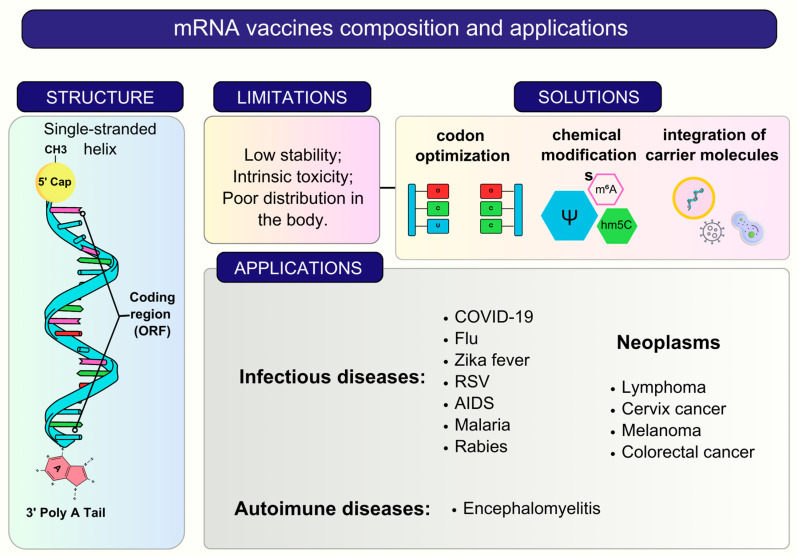
A general summary of composition, features, and applications of mRNA vaccines. The cap (5′) and a poly-A tail (3′) flanking the ORF are required for the translation of target proteins and represent the typical features present in the structure of mRNA vaccines. These vaccines, however, have drawbacks such as low stability, inherent toxicity, and poor distribution in the body. Codon optimization, chemical changes, and the usage of carrier molecules are being employed to solve these issues. For medicinal applications, mRNA vaccines have demonstrated promise in treating infectious, neoplastic, and autoimmune illnesses, opening the path for clinical improvements.

The mRNA vaccines comprise synthetic single-stranded molecules that direct the translation of the antigen responsible for activating the immune response. Like endogenous mRNA, the in vitro assembly includes one or more antigen-encoding ORFs, untranslated regions, a 3′ poly-A tail, and a 5′ methylated cap that prevents unintended immune responses and degradation [5,20] (Figure 1).

Current classifications include three main types of RNA vaccines: conventional (mRNA), without modifications and self-amplifying capacity; base-modified non-amplifying mRNA (bmRNA) vaccines; and auto-amplifying mRNA (saRNA) vaccines [21]. RNA vaccines must enter the cytoplasm of antigen-presenting cells, which are translated and processed for presentation via MHC-I and subsequent activation of CD8+ T cells [22]. On the other hand, synthesized proteins located in the extracellular milieu can be captured, degraded, and presented via MHC-II, causing the activation of CD4+ T cells critical for the activation of defense cells and antibody production [23]. However, due to drawbacks such as stability, inherent toxicity, and poor distribution, RNA vaccine applicability was initially restricted compared to other platforms [3]. To overcome these issues, the application of chemical modifications, codon optimization, and incorporation of carrier molecules has broadened their usefulness and sparked significant interest in the production of vaccines against infectious [5], autoimmune [24], and neoplastic diseases [25] (Figure 1). Despite advancements in their production and modification, RNA vaccines still face several challenges, with thermal stability being a major concern. 

Unlike most traditional vaccines, mRNA vaccines are temperature sensitive, which can reduce their efficacy. RNA is inherently unstable, and external factors like temperature, pH, and enzymatic activity can cause it to degrade quickly [3,26]. Furthermore, if proper stabilizing agents are absent, mRNA molecules may be more susceptible to immune recognition and clearance before reaching their target cells [5,27]. As a result, researchers have been looking into ways to improve the thermal stability of mRNA vaccines. One strategy involves using modified nucleosides to prevent recognition by pattern recognition receptors (PRRs), lowering the immune response and subsequent degradation. Most RNA vaccines include modified nucleosides to avoid recognition by PRRs and ensure sufficient translation of the target antigen [28]. Unaltered mRNA molecules are highly immunogenic, identified by Toll-like (such as TLR3, TLR7, TLR8, and TLR13), RIG-1, and MDA-5 receptors, and capable of inducing cellular and humoral responses, although some modifications are required [29]. However, they can cause the synthesis of immunostimulatory molecules such as type 1 interferons (IFN-I), which may hamper the translation of the antigenic protein [30].

The success of mRNA vaccines also depends on delivery techniques. The vehicle must protect the RNA from possible digestion by ribonucleases and allow for effective uptake by the target cell, dissociation of genetic material from the delivery vehicle, and escape from endosomes [21,31]. In addition, non-toxicity and immunostimulation are imperative for an ideal delivery vehicle [27]. For example, lipid nanoparticles in Moderna and Pfizer’s mRNA vaccines against COVID-19 provide delivery, protection, and activation of the innate immune response [4,32]. However, they must be stored at low temperatures, necessitating a cold chain along the distribution route, since there is a possibility of decreased efficacy and instability due to mRNA hydrolysis [33]. Moreover, despite the preferential study by delivery particles of lipid and polymeric origin, new approaches involving natural delivery systems based on microorganisms emerge as novel systems to be explored [31,34,35]. The main systems for the delivery of RNA vaccines are summarized in Table 1.

Some microorganisms, such as yeasts, have demonstrated promise as biofactories for therapeutic proteins and carriers of biological molecules [8,10]. These natural delivery systems promote efficient immune responses, specific uptake, and transport of nucleic acids anchored superficially or in their interior [8]. However, further research is needed to evaluate their applicability in mRNA vaccine delivery and understand the mechanisms of enhancing vaccine effectiveness and distribution.

## 3. Yeasts as Vaccine Carriers: Characteristics and Immunological Aspects

Besides being recognized biotechnological platforms for the obtaining of immunobiological products, yeasts such as *Saccharomyces cerevisiae* and *Pichia pastoris* have been proposed as delivery systems for protein antigens or nucleic acids in the development of prophylactic and therapeutic vaccines [53]. This approach, called whole yeast vaccines, allows for better delivery and antigen presentation to the immune system through intracellular delivery or even anchoring recombinant proteins on the yeast cell surface [8,9,54]. The recombinant yeasts are heat inactivated in the whole yeast vaccines (WYV) to ensure safety. So far, adverse effects or toxicity related to the administration of yeasts in an animal model and humans have not been reported. The safety of these species is certified by the GRAS status (Generally Regarded As Safe). Most studies with yeast-based vaccines are in the preclinical phase, using several animal models such as mice, fish, pigs, and chickens [53]. Although WYV is not yet commercially available, some therapeutic vaccines for cancer and chronic diseases are already in the clinical evaluation phase [17,18,19,34]. The WYVs in clinical studies are part of the Tarmogen platform (GlobeImmune), such as GI-6207, GI-4000, GI-6301, and GS-4774 patented strains. The stability of the yeast-based vaccines has been evaluated, exhibiting viability even after one year both under refrigeration (2–8 °C) and at room temperature (23–25 °C) [55]. Some conventional vaccines lose their antigenic capacity and undergo degradation when the cold chain breaks, which limits their distribution in low-resource countries [56]. On the other hand, lyophilization of recombinant yeasts used as whole yeast vaccines allows storage at room temperature, preserving the antigenic capacity of the vaccine antigen for up to one year. This thermal stability was evaluated upon storage at 30 °C and 37 °C for more than one year, and it represents an advantage for establishing these organizations as a delivery platform for nucleic acid-based vaccines [53,57].

**Figure 2:**
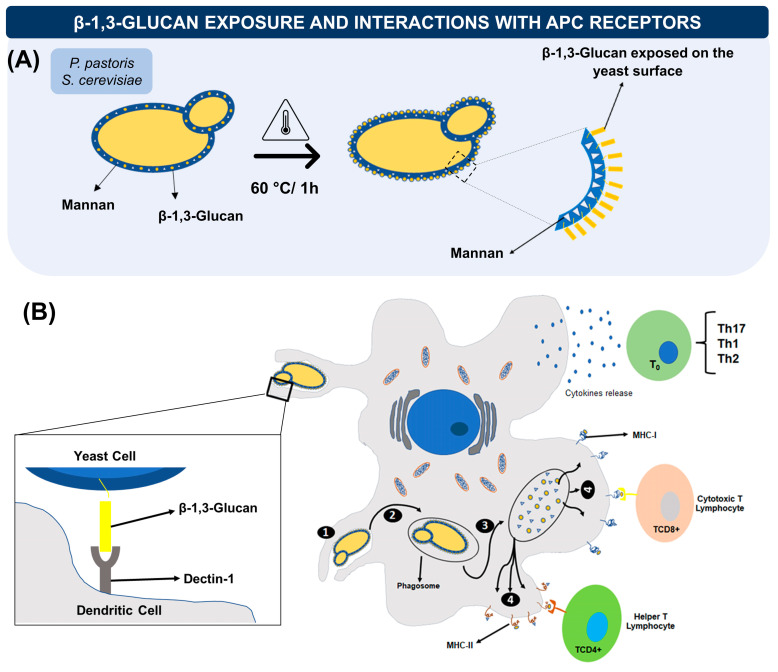
(**A**) Heat treatment promotes an increase in the exposure of β-1,3-glucans on the yeast surface (heating at 60 °C for at least 1 h), enhancing the adjuvant capacity of the yeast, favoring its recognition and binding to receptors present in antigen-presenting cells. (**B**) Dendritic cells recognize yeast β-1,3-glucans mainly through the Dectin-1 receptor that promotes and facilitates phagocytosis (1). After the uptake, the phagosome is formed, where the yeasts are processed. The fragmented antigens can be presented by MHC-I (cytosolic proteolysis) leading to the activation of cytotoxic TCD8+ lymphocytes, or by MHC-II (endolysosomal proteolysis) inducing the activation of TCD4+ Helper lymphocytes. (2) Inflammatory cytokines such as TNF-α, IL-6, IL-8, and IL-1β or IL-12, IL-23, and IL-27, released by activated dendritic cells polarize T cells to Th1 and Th17 profiles (3).

An advantage of the use of yeasts as vaccine vehicles is their immunostimulatory property due to the presence of β-1,3-glucans and mannoproteins in their wall, that, when detected by Pattern Recognition Receptors (PRRs), promote the release of cytokines, chemokines, and eicosanoids that modulate the inflammatory response in addition to leading to the development of specific adaptive immune responses against the pathogen [58,59,60]. As with mannoproteins, the response induced by β-1,3-glucans tends towards a pro-inflammatory Th1 profile. Besides, these particles can promote the recruitment of neutrophils and specific antigenic recognition by APCs when interacting with receptors such as Dectin-1 [61]. For some species as *S. cerevisiae* and *P. pastoris*, the exposure of β-1,3-glucan can be favored by heating the yeast at 60 °C for at least 1 h, expanding their adjuvant property [62] (Figure 2A).

Immunostimulation induced by yeasts may be greater than that induced by classic adjuvants such as aluminum salts [63]. Unlike yeast, aluminum salts used as adjuvants in vaccines can generate adjuvant-induced autoimmune/inflammatory syndrome (ASIA) in the applied area, promoting inflammation [64,65,66]. In addition to the inflammatory process, the high recruitment of neutrophils to the inflamed area seems to interfere with antigen presentation by DCs and macrophages. Moreover, aluminum salts generate an immune response mediated by Th2 cells, which may interfere with the efficiency of responses expected for vaccines developed against chronic infectious diseases and cancers [67,68].

In dendritic cells (DCs), the capture and phagocytosis of yeasts are mediated by the presence of the Dectin-1 receptor that recognizes the β-1,3-glucans. Among the different receptors responsible for yeast recognition and phagocytosis, Dectin-1 is the receptor on APCs that recognize β-glucans, the major component of the yeast cell wall [69,70] (Figure 2B). Activation of DCs by yeast promotes the secretion of inflammatory cytokines such as TNF-α, IL-6, IL-8, IL-1β, IL-12, IL-23, and IL-27 that polarize T cells for Th1 and Th17 [71]. This polarization is particularly interesting in the context of immunotherapies against viral infections or anticancer, reinforcing the response induced by the vaccine antigen carried by the yeast.

## 4. Biodelivery of mRNA Vaccines by Yeasts

**Figure 3:**
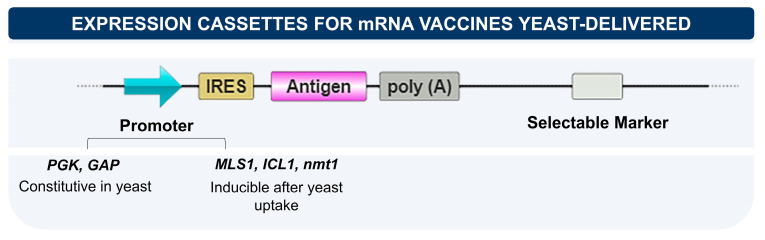
Main components of expression vectors used to carry mRNA vaccines by yeast. The choice of the promoter, constitutive or inducible, may influence the successful transcription of the target gene (also depending on the phagocytic cell involved, macrophage, or dendritic cell). The IRES sequence (derived from EMCV) prevents the translation of vaccine antigens from occurring within yeast cells before phagocytosis. In addition to these components, the poly(A) sequence is important for stabilizing mRNA. It is necessary to add a marker, preferably by auxotrophic, to select recombinant yeasts.

The ability to use yeast as a vaccine vector has been explored in different studies for the therapy of different types of cancer, as immunomodulators, and for the treatment or prophylaxis of infectious diseases [15,17,72]. In this context, yeast can also be used to carry RNA molecules. For this, it is necessary to use vectors with components for adequate expression and delivery of the genetic material to the target cells or tissues (Figure 3). For mRNA delivery, plasmids with promoters derived from yeast are chosen to allow transcription of the gene of interest before or after phagocytosis by APCs [10]. One of the most adopted vectors is the *PGK1*, derived from the phosphoglycerate kinase (PGK) pathway. As well as *PGK1*, other constitutive promoters such as *GAP* (glyceraldehyde-3-phosphate dehydrogenase) mediate transcription before the yeast phagocytosis [71]. On the other hand, it is possible to employ inducible promoters, such as malate synthase (*MLS1*), *nmt1* (thiamine-repressible) and isocitrate lyase (*ICL1*), whose activation and transcription mediation happen just after phagocytosis [15,73]. Furthermore, another inducible promoter that can be exploited is alcohol oxidase I (*AOX1*), commonly used to regulate the expression of heterologous proteins in *P. pastoris* [74,75], as well as the *GAL* promoter that is inducible by galactose and can have its expression reduced or increased, depending on the carbon source that the yeast is using, glucose or ethanol [76].

The simultaneous carrying of DNA and RNA vaccines by yeast is a promising strategy. In this case, the vector used for yeast transformation must present a promoter for each vaccine antigen [72]. In addition to a yeast promoter for RNA vaccines, it is necessary to include a promoter functional in mammalian cells suitable for DNA vaccines. In this case, the default promoter is CMV, derived from Cytomegalovirus [10]. The combined nucleic acid vaccine, yeast-carried, is effectively expressed and can be successfully delivered for immunized animals [72].

Another component of the expression cassette, upstream to the target gene, is an IRES (Internal Ribosomal Entry Site) sequence derived from EMCV (encephalomyocarditis virus). This region prevents antigen translation in yeast cells and increases translational levels in host cells, mainly in the macrophages [11,73]. The influence of the IRES sequence on the expression cassette was demonstrated in in vitro studies using the IC-21 murine macrophage cell line, confirming the absence of translational activity in yeast and the translation of the antigen model in mammalian cells after phagocytosis [11].

One of the main applications of mRNA delivery by yeast has been immunomodulation in therapeutic approaches [77]. Polarization of tumor-associated macrophages (TAMs) to an M1 expression profile has been used in several cancer immunotherapy strategies [28,78]. The carrying of genes encoding pro-inflammatory cytokines, such as TNF-α, by *S. cerevisiae* may be a useful tool for altering the profile of these TAMs within the context of the tumor microenvironment [15]. In addition to the isolated use of RNA, new studies have emerged applying yeast to carry multiple strategies in the same expression cassette.

## 5. Capsules, Microparticles, and Nanoparticles of Yeast β-glucans for RNA Delivery

**Figure 4:**
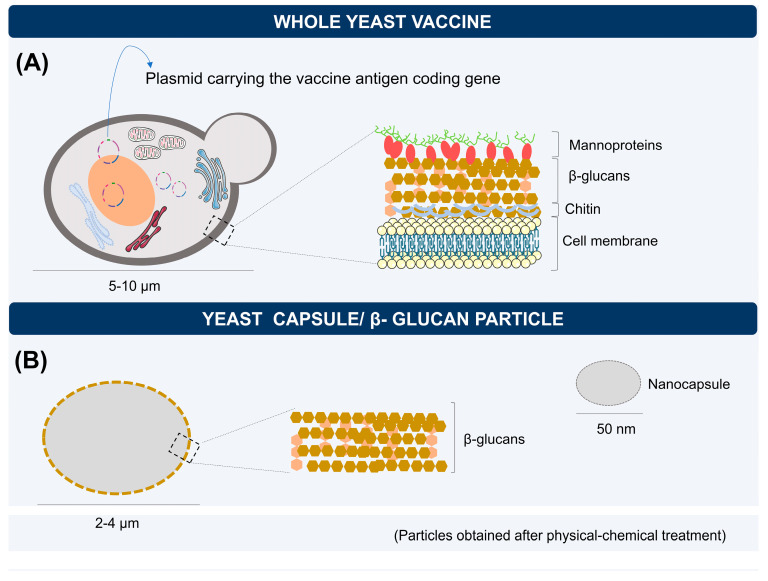
Yeast-based vaccine modalities. (**A**) Yeast, as a complete organism, can carry plasmids with the desired antigen gene inside them. The immunostimulatory effects result from the interaction between the yeast cell wall components and macrophage and dendritic cell receptors. (**B**) β-glucan microcapsules or nanocapsules are particles obtained after physical-chemical treatment to remove the mannoprotein layer. Differences in size between the whole cell and the β-glucan particles can influence the phagocytosis process and the delivery and dissemination of antigens through lymphatic vessels.

The yeast-based biodelivery goes beyond using the whole cell as a vaccine vehicle. Yeast cell particles (YCP) obtained from the mannoprotein layer removal have also been used for this purpose [12,79,80] (Figure 4). These particles can vary in size and be classified as capsules, microparticles, or nanoparticles depending on the treatment conditions used to allow access to the β-glucans layer [27,81,82]. The association with other immunostimulants such as aluminum salts [83] and formylate [84] or the introduction of chemical modifications can further improve the function of glucan particles (GPs) as a vaccine adjuvant, offering an interesting method to trigger the vaccine immune responses [85].

The capsules can be recognized as a pathogen-associated molecular pattern (PAMP), induce and modulate immune responses, and can carry proteins, DNA, RNA, and small molecules. Antigen delivery based on these particles allows oral administration and promotes antigenic targeting to APCs due to the presence of β-1,3-D-glucan receptors in these immune cells [14,84,86,87]. These receptors include Dectin-1, complement receptor 3 (CR3), and Toll-like receptors (TLRs). Once phagocytosed, GPs can mediate the stimulation of components of innate immunity, such as macrophages, dendritic cells, neutrophils, and NK cells, and lead to the secretion of pro-inflammatory cytokines [84,88].

GPs are 2–4 µm hollow and porous structures that allow the encapsulation of a high load of antigens through central polyplexes and synthetic structures that help maintain the conformation and integrity of the particles for targeted delivery of antigens to APCs [85]. GPs as delivery systems for nucleic acids have demonstrated protection and ease of oral delivery targeting specific components of mucosal immunity, besides being able to promote systemic immune responses [87]. Vaccine formulations based on GP nanoparticles (NPs) offer significant potential for new vaccination strategies due to their unique physicochemical properties and the size of these structures that facilitate their uptake by dendritic cells [84]. An additional application of β-glucan particles is to coat their surfaces with nanoparticles carrying drugs or vaccine antigens to target cells or specific tissues, as tumor-associated macrophages that are interesting targets for immunotherapy approaches against cancers [85,89].

The delivery of siRNA by GP NPs demonstrates high intracellular delivery capacity, even when compared to commercial carriers such as Lipofectamine, and has low toxicity [90].These NPs can stably and efficiently carry antigens, mediate the delivery of these antigens, and enhance maturation, stimulation of DCs, and the secretion of pro-inflammatory cytokines [91].

GPs lack reproduction activity and are smaller than whole yeast cells [92]. The morphology of these particles, especially nanoparticles smaller than 50 nm, facilitates their entry through lymphatic vessels, being more easily absorbed by DCs, while large glucan particles are mainly delivered to lymph nodes in a cell-mediated manner [92,93]. Furthermore, vaccines that use whole organisms contain a wide range of antigens and, although they are relatively easy to manufacture, may present concerns about reactogenicity, autoimmunity, and possible infection in immunosuppressed populations when administered [88,92,94]. The use of particles derived from the cell wall of yeasts has been shown to be an option to circumvent any adverse effects arising from the administration of whole microorganisms. However, more comprehensive in vitro and in vivo evaluations are needed to address the immunotoxicity and bioavailability of these vaccine carriers.

## 6. Routes of Administration Using Yeast for Delivery of Vaccines

When assessing the efficacy of a vaccine strategy, the route of administration is an essential factor in immunogenicity as it determines effective antigen presentation, potency, and effector response [95]. Thus, investigations on the invention and application of techniques that increase vaccine distribution have been conducted, with the overall conclusion that adjuvants and delivery systems are required [8]. Alternatively, the yeast-based distribution technique, whether in whole yeast, capsules, microparticles, or nanoparticles, is a valuable alternative, particularly for genetic vaccines emphasizing RNA, as discussed in previous sections of this paper.

Oral, subcutaneous, intramuscular, and administration are common routes for yeast-based vaccines (Table 2). The intravenous route results in systemic dissemination, or yeast migration, to organs such as the heart, kidney, spleen, and liver. However, in the study by Becerril-García et al. (2022), it was found that, despite being circulating, there was a rapid elimination of the yeast *P. pastoris* administered intravenously in mice (about 48 h after its inoculation) since the absence of pathogenicity and ability to colonize leads to its elimination from the organism. Furthermore, it did not show the capacity to induce the secretion of cytokines and immunoglobulins, such as TNF-α, IL-6, IgM, and IgG, or a cellular response in the serum of the inoculated animals. Despite this, this lack of response to the vector minimizes the chances of adverse events occurring because of the innate immunity reaction against the yeast. The results showed no pathologies or alterations in the body, highlighting its safety [96].

Regarding the intramuscular route, the currently studied vaccines use a portion of the structures found in the cell wall of yeasts known as B-glucans [97,98]. These compounds bind to cell receptors, activating immune response signaling pathways [99]. As a result, they are classified as adjuvant molecules [97,100,101]. Vaccine strategies containing yeast B-glucans administered intramuscularly have not yet been developed for use in humans (Table 1). Wang et al. (2014) used a version of this component derived from the *Saccharomyces cerevisiae* cell wall with sulfate to treat Newcastle disease (ND) in chickens [98]. They discovered that birds receiving the sulfated B-glucan vaccine increased in vitro splenocyte proliferation and in vivo IL-2, IFN-Y, and serum antibody production. Diao et al. (2013) investigated the sulfated version in fish with glyceraldehyde-3-phosphate dehydrogenase (GAPDH). Compared to fish in the control group, which received only GAPDH, those immunized with these two compounds had a higher survival rate, transcription level of immunomodulators, and antibody production [97,101].

Another typical route is subcutaneous administration, explored in the preclinical and clinical phases. Bilusic et al. (2014) used the yeast *S. cerevisiae* to express human carcinoembryonic antigen (CEA) as a therapeutic strategy against cancer. Patients received the vaccine subcutaneously every two weeks for three months [17]. The results indicated low toxicity, increased antigen-specific T cells, and CEA stability. In another study focused on neuropathology by Liu et al. (2020), a yeast-based vaccine exhibiting beta-amyloid (Aβ) on its cell surface was applied subcutaneously in mice. The vaccine was able to induce antibodies against Aβ and contribute to the improvement of the cognitive capacity of the immunized animals [102].

While vaccines are commonly administered through intramuscular or subcutaneous routes in numerous studies, these traditional methods have limitations. They present challenges in achieving a consistent and specific cellular and humoral response, providing inadequate protection in mucosal areas, and causing discomfort during the application, all contributing to low adherence rates [103]. On the other hand, oral administration is favorable, allowing systemic immunostimulation and mediation on mucosal surfaces, leading to an adaptive response that combines the action of IgA antibodies and resident memory T cells [104]. This last feature results in specific and efficient protection since most pathogens have mucosal areas as their gateway. In addition, the oral route is preferable because of its low cost of production and ease of distribution [51].

Despite this, some hurdles must be overcome in oral vaccines since natural local barriers, such as those found in the gastrointestinal tract, make accessing and distributing vaccine components problematic, resulting in limited antigen availability. Physical structures such as the mucus and glycocalyx layer, as well as low pH and the presence of proteases and nucleases on the mucosal surface that cause the breakdown of nucleic acids and antigenic proteins included in the formulation are among the reasons [51,105].

Alexander (2021) describes how yeast-based delivery systems for oral gene delivery can be explored both as vaccine strategies and for immunotherapy. Furthermore, the antigen can be delivered to the target tissue via the intestinal mononuclear phagocytic system, which then transports it to the systemic circulation [51]. In this context, for RNA strategies, for example, it is necessary to use pathways that keep the antigen safe and stable until it reaches target tissues and cells, allowing recognition and robust response by the immune system [10]. Therefore, the route chosen will depend on the type of approach and the suitable characteristics for each strategy (Table 3).

**Table 2:** Examples of vaccines delivered by yeast in different routes of administration.

Route	Type of Antigen	Vehicle	Animals	Main Findings	Ref.
	Tnf-α and Map4k4 (siRNA)	Yeast capsule	Male C57BL6/J mice	The orally absorbed Map4k4-siRNA-containing GeRPs underwent siRNA-mediated gene silencing and protected mice from LPS/d-GalN-induced lethality by inhibition of TNF-α and Il-1β production in macrophages.	[106]
Oral	CD40 (shRNA)	Whole yeast	Female C57BL/6 mice	The shRNA carried by yeast effectively repressed the target gene (CD40) in vivo and had a significant effect on IL-6, IL-10, IL-12, and TNF-α expression.	[16]
	IL-1β (shRNA)	Yeast microcapsule	Male C57BL/6 mice	Yeast microcapsule-mediated the delivery of IL-1β shRNA successfully, downregulated the intestinal inflammatory response in PTOA mice.	[82]
	miR365 antagomir	Yeast cell wall particle (YCWP)	Male C57BL/6 mice	The results showed that NPs-YCWP can effectively resist the corrosion of SGF, and was successfully engulfed by macrophages.	[107]
	rH9- DNA- RNA	Whole yeast	Chicken	Both DNA and RNA cassettes were successfully delivered by yeast and the vaccines elicited humoral and cellular immune responses.	[72]
I.V.	-	Whole yeast	Female C57BL/6 mice	The results demonstrate that after *P. pastoris* inoculation, there was no pathology in the tested mice, and its dissemination to some tissues is quickly eliminated in the first hours. Thus, its use was considered safe for the development of vaccines, highlighting the intravenous route.	[96]
	GI-5005 (HCV NS3-Core)	Whole yeast	BALB/cBy and C57BL/6J mice	The immunization with GI-5005 led to the induction of cytotoxic effector cells that can kill syngeneic tumor cells expressing NS3.	[108]
S.C.	Yeast-CEA	Whole yeast	Female C57BL/6 mice	The study showed that the vaccine reduces tumor burden, and extends overall survival in CEA-transgenic mice. Furthermore, it was able to elicit increased antigen-specific T-cell responses after each vaccination.	[109]
	GI-6301 (Yeast-Brachyury vaccine)	Whole yeast	Adults with advanced or metastatic chordoma (Phase I)	The vaccine was safe and immunogenic in humans. And two chordoma patients showed evidence of disease control (one mixed response and one partial response).	[110]
	Y-5A15 (Yeast/Aβ1-15)	Yeast surface display	APP/PS1 transgenic AD mice	The vaccine exerted favorable effects on cognition and neuropathology in the mice. Furthermore, it induced high titers of antibodies against Aβ.	[102]
I.M.	Sulfated B-1,3–1,6-glucan from *S. cerevisiae*	Yeast cell wall particle modified	Chicken	The results showed increases in the levels of splenocytes, IL-2, IFN-Y and serum antibodies.	[98]
	B-1,3–1,6-glucan with glyceraldehyde-3-phosphate dehydrogenase (rGAPDH)	Yeast cell wall particle modified	Fish	The results showed increases in survival rate, level of transcription of immunomodulators and production of antibodies.	

shRNA (short hairpin RNA); siRNA (short interfering RNA); GeRPs (β1,3-d-glucan-encapsulated siRNA particles); Tnf-α (tumor necrosis factor α); IL-1β (interleukin-1β); miRNA (microRNA); SGF (Simulated gastric fluid); CEA (Human carcinoembryonic antigen); rGAPDH (glyceraldehyde-3-phosphate dehydrogenase recombinant with B-1,3–1,6-glucan). Route: I.V: Intravenous; S.C.: Subcutaneous; I.M.: Intramuscular.

## 7. Delivery of RNA Interference (RNAi)

In addition to mRNA-based vaccines, different experimental models have demonstrated that it is possible to silence or modulate the expression of target genes in a specific way in eukaryotic cells through dsRNA (double-stranded RNA) or shRNA (short hairpin RNA) molecules [111,112]. These interference RNAs compose a regulatory pathway that leads to the silencing of genes due to sequence homology and complementarity associated with an enzymatic complex that promotes target mRNA degradation [113,114].

In recent years, the understanding of RNAi has enabled its use as a gene therapy tool against infections caused by different viruses such as coronaviruses associated with severe acute respiratory syndrome, influenza, hepatitis B virus, and Dengue virus [90,115,116,117]. In addition to controlling these infections in their acute phase, this gene-silencing mechanism has also been used in therapeutic strategies aimed at cancer and chronic inflammatory diseases [118,119]. RNAi molecules (siRNA, shRNA, miRNA) can be the main component of therapeutic vaccines aimed at immunomodulating immune cells or silencing targeting genes. Furthermore, gene therapy based on RNAi can complement other immunotherapies, seeking a synergistic effect and enhancing the desired immune response [120].

However, the success of this strategy involves both the effectiveness of silencing and the non-occurrence of toxic effects. Thus, some adaptations are performed, including chemical modifications and changes in size, charge, and hydrophilicity that can interfere with the cellular uptake and biodistribution of RNAi molecules [121]. The administration method and the particle size of the vehicle influence the bioavailability and biodistribution of the loaded RNAi.

In this sense, the efficiency of this approach also depends on the use of efficient delivery systems [16,121]. Liposomes, bacteria, and viruses are examples of platforms used as vehicles for interfering RNAs, as already used for DNA and mRNA vaccines [38,47,122]. However, some limitations indicate the need to seek alternatives to these approaches. Among these limitations are certain restrictions on the carrying capacity, besides issues related to safety and induction of immune tolerance or neutralization of the vaccine vector [123].

In contrast, the yeasts in their various presentations (whole cell, microcapsules, capsules) are feasible vehicles for the protection and specific delivery of therapeutic vaccines based on shRNA or dsRNA [16,123,124]. Antigen delivery remains efficient even if the yeasts undergo heat treatment to eliminate their reproductive activity and are freeze-dried and processed in the form of capsules or tablets [125,126]. The therapeutic interfering RNA can be eliminated from the body in hours or days, even if it is nanoparticulate. The reduced bioavailability time may impair the immunotherapeutic product reaching the target cells or tissues [121]. Otherwise, the durability of gene silencing was already demonstrated, even 50 days after oral administration of shRNA carried by *S. cerevisiae*, as observed by analyzing the dendritic cells from the intestine of immunized animals [16]. Although this study was carried out in an animal model, it shows promising effects regarding the duration of the immune response by the proposed therapy, that may be evaluated in humans.

After oral administration, the yeasts are phagocytosed by macrophages and dendritic cells in the gut-associated lymphatic tissues (GALT). These phagocytic cells, which are APCs, can migrate and infiltrate other lymphatic tissues and induce specific responses even in sites distant from the point of administration [82]. This mechanism was observed by Zhang et al. (2021), evaluating the delivery of an shRNA for silencing the IL-1β cytokine as a therapeutic strategy for post-traumatic osteoarthritis. In this study, to access the eventual tissue-specific migration of phagocytosed yeasts, yeast cells were labeled with near-infrared fluorescence, allowing the in vivo evaluation of the delivery of biomolecules to verify the specificity of this delivery and the induction of immunological response at the target sites. Such marking allowed observing the presence of macrophages carrying the recombinant yeasts in the joint and knee regions of the studied mice [82,127].

As already mentioned, the size of the yeast and the binding to specific receptors such as Dectin-1 and CD206 favor the process of phagocytosis by antigen-presenting cells [69]. These immune cells are appropriate targets for RNAi-based therapies as they mediate the chronic inflammatory response caused by certain clinical conditions and autoimmune diseases [128]. This type of specificity is also essential to minimize toxic effects that could result from silencing in inappropriate cells or tissues.

In therapies based on RNAi transport by yeast, the main targets explored for silencing and immunomodulation are cytokines that play an effector role in the differentiation or maturation of APCs (e.g., IL-1β and IL-21), cellular receptors (e.g., CD40), repressor molecules (e.g., indoleamine 2, 3-dioxygenase—IDO), or even specific antigens of infectious agents [14,16,82,129]. Zakria et al. (2019) developed an interesting study using *S. cerevisiae* as a vehicle for a vector designed for a DNA vaccine encoding the myostatin gene and for delivery of an IL-21 shRNA vaccine as therapeutic targets. Studies like this open the way for improving vaccines with more than one type of vaccine antigen that can complement or amplify the desired immune response [130].

**Figure 5:**
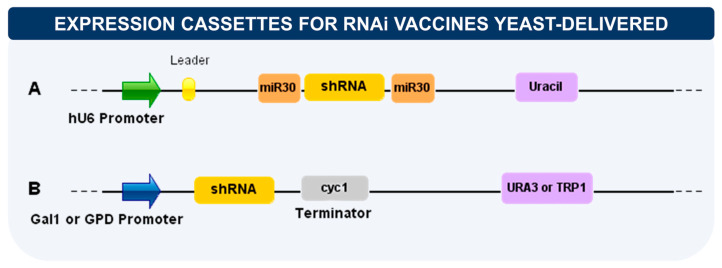
Main elements present in expression vectors for RNAi delivery. (**A**) Cassette with a promoter recognized by mammalian cells. The cassette should also contain a leader sequence just after the promoter, and it is recommended to flank the target gene shRNA by endogenous miRNA (e.g., miR30). Uracil is the selection marker usually employed. (**B**) Cassette for expression with yeast promoters (constitutive or inducible). URA3/TRP1 sequences are loci for cassette integration and auxotrophic marker genes.

In addition to selecting therapeutic targets, it is necessary to consider how to construct the expression cassettes to be inserted into the yeast. Species like *S. cerevisiae* have a vast set of expression vectors that can be adapted for this type of application. The main components of these cassettes are the promoter, the selection mark, and the integration sites if desired. Both episomal and integrative vectors have been used, and integration has been seen to ensure more stable delivery. However, how this stability and the number of RNAi copies per cell can influence the effect or duration of gene silencing is still unclear [123,125]. Concerning the selection of recombinant yeasts containing the expression vectors, it is preferable to use auxotrophic selection marks such as URA3/TRP1 that minimize the use of antibiotics and the possible horizontal transfer of resistance genes. These vectors are liable to optimization and vary according to the strategy (Figure 5). Regarding transcriptional control, promoters functional in mammalian cells as hU6 [95] or active only in yeast as GDP1 (constitutive) and Gal1 (inducible) can be used [123]. When using a promoter such as hU6, transcription must occur only after yeast phagocytosis and the release of its intracellular content where the recombinant plasmids are.

On the other hand, when using adopted native yeast promoters, it is expected the transcription and accumulation of RNAi in the yeast cell, such as *S. cerevisiae*, do not express Dicer and Argonaute. Thus, the bioengineered RNAi molecules can be accumulated without degrading [123,124]. It is worth pointing out that this characteristic is not common to all yeast species and strains that must be considered in preparing vaccines with yeast-delivered RNAi [130]. It is also important to point out that the type of promoter chosen (inducible versus constitutive) can also impact the scaling of cultures to prepare vaccine doses [131]. Induction of gene expression by adding certain compounds can be unfeasible for producing yeast-based vaccines on an industrial scale [53,123].

## 8. Conclusions

The development of RNA vaccines has gained a greater dimension due to the addition of optimizations in its structure and the discovery of new carriers capable of protecting the molecule, allowing their specific uptake to the target cells. Yeast cells or their derivatives (β-glucan particles) have these features appropriated for a vaccine delivery system besides their use as immunomodulators in therapeutic strategies. Despite the mentioned advantages, some points of study and investigation remain open for wide employment of yeast-delivered RNA vaccines. Appropriate methods for quantifying the carried RNA are still not well established, as well as the most effective protocols for incorporating RNA vaccines in yeast (whole yeast vaccines) and β-glucan capsules. To date, most of the published studies involve proofs of concept evaluated in a preclinical model. The analysis of the results of preclinical studies regarding the quality and safety of RNA vaccines carried by yeast indicates that in the medium term, these vaccines will advance to clinical phase studies. However, it is still necessary to obtain results from clinical studies evaluating the therapeutic action of this approach, to establish methods for quantifying yeast-delivered nucleic acids, standardize the concentration parameters of doses, and even to explore more yeast species that can be used (beyond *S. cerevisiae*). In addition, most of the thermal stability and duration of vaccine antigen expression studies have been tested for yeasts carrying protein antigens. Such adjustments are essential for yeast-delivered RNA vaccines to reach the pharmaceutical market. 

## Figures and Tables

**Table 1:** Comparison among the delivery systems explored for RNA vaccines.

Delivery System	Advantages	Disadvantages	References
Lipid-Based	- High transfection efficiency - Effective delivery of various RNA types - Enhanced stability and protection of RNA - Suitable for large-scale production - Tissue-specific targeting	- Potential cytotoxicity and inflammation - Risk of rapid clearance from circulation - Some formulations may require cold storage - Batch-to-batch variability - Risk of accumulation in certain organs	[36,37,38,39]
Polymer-Based	- High biocompatibility - Controlled release of RNA - Potential for sustained RNA delivery - Versatile for RNA modification - Less immunogenic than viral vectors	- Lower transfection efficiency compared to viral vectors and some lipid-based systems - Complex and laborious synthesis process - Risk of toxicity depending on the polymer used - Limited cargo capacity - Possibility of aggregation and instability	[40,41,42,43,44]
Viral Vectors	- Highly efficient RNA delivery - Long-lasting gene expression - Suitable for gene therapy applications	- Risk of insertional mutagenesis - Potential immunogenicity and toxicity - Difficult to scale up production	[45,46,47]
Bacterial Vectors	- Natural affinity for certain tissues - Large cargo capacity - Ability to target specific cells - Potential for non-invasive delivery	- Risk of inducing immune response - Possibility of genetic modification - Safety concerns due to potential pathogenicity - Limited availability of suitable bacterial vectors	[48,49,50]
Yeast	- Generally regarded as safe (GRAS) - Non-toxic and biodegradable - Allow repeated administration without vector neutralization - Easy and cost-effective scale-up - Can target specific tissues/cells	- Possibility of batch-to-batch variability - Lack of standard protocols for antigen quantification	[10,11,51,52]

**Table 3:** Some advantages and limitations of yeast-based vaccines by different routes of administration.

Routes	Advantages	Limitations
Oral	- Promotes systemic immunostimulation - Promotes mucosal immunity - Action of IgA antibodies and resident memory T cells - Low cost of production - Resistance to the gastrointestinal tract barrier	- Protein degradation (strategy of Yeast Surface Display) - Amount of yeast cells in the vaccine preparation
S.C.	- Low toxicity - Good stability - Production of specific T cells	- Variable and nonspecific cellular and humoral response - Low induction of protection in mucosal regions - Low adherence (local discomfort)
I.V.	- Systemic dissemination - Fast effect - Large volume administration	- Rapid elimination of yeast after inoculation - Low induction of cellular response - Increased risk of infections by contamination
I.M.	- Good induction of antibody production - Relatively fast absorption - Moderate volumes	- Low induction of protection in mucosal regions - Low adherence - Local discomfort

## Data Availability

Not applicable.
